# A Novel Pyroptosis-Associated Gene Signature to Predict Prognosis in Patients with Colorectal Cancer

**DOI:** 10.1155/2022/6965308

**Published:** 2022-05-17

**Authors:** Juji Dai, Shuangdong Chen, Yongyu Bai, Yangyang Fu, Yifei Pan, Lechi Ye

**Affiliations:** ^1^Department of Colorectal and Anal Surgery, The First Affiliated Hospital of Wenzhou Medical University, Ouhai District, Wenzhou, Zhejiang 325000, China; ^2^Department of Anesthesiology, The First Affiliated Hospital of WenZhou Medical University, Wenzhou, Zhejiang 325000, China; ^3^Department of Gastrointestinal Surgery, The First Affiliated Hospital of Wenzhou Medical University, Wenzhou, Zhejiang 325000, China; ^4^Division of Pulmonary Medicine, Key Laboratory of Heart and Lung, The First Affiliated Hospital of Wenzhou Medical University, Wenzhou, Zhejiang 325000, China

## Abstract

**Background:**

Pyroptosis is a form of cell death characterized by cell swelling and plasma membrane bubbling in association with inflammatory and immune responses. To date, the association between pyroptosis and colorectal cancer remains unclear. We aimed to establish a novel pyroptosis-associated model for the prognosis of colorectal cancer.

**Methods:**

Pyroptosis-related genes were extracted using Gene Set Enrichment Analysis. A least absolute shrinkage and selection operator regression model was constructed to identify a pyroptosis-related gene signature using the Cancer Genome Atlas and Gene Expression Omnibus databases. Then, Kyoto Encyclopedia of Genes and Genomes and Gene Ontology and GSEA were performed to better understand the potential mechanisms and the functional pathways associated with pyroptosis involved in colorectal cancer. The relationship between the pyroptosis-related signature and immune infiltration was investigated using Cell-Type Identification by Estimating Relative Subsets of RNA Transcripts and MCPcounter.

**Results:**

A 12 pyroptosis-related gene signature was identified. Then, patients were classified into high- and low-risk groups. Kaplan–Meier and receiver operating characteristic analyses confirmed that the high-risk groups showed worse overall survival, progression-free survival, or relapse-free survival probability. Functional enrichment analysis showed that pyroptosis was associated with extracellular matrix-related pathways. Furthermore, the pyroptosis risk score was associated with immune infiltration. The low-risk group exhibited a higher percentage of plasma cells, CD4 T cells, activated dendritic cells, and activated mast cells. M2 macrophages and M0 macrophages were positively related to the risk score.

**Conclusion:**

Our research yielded a novel pyroptosis-related prognostic signature for colorectal cancer that was related to immune cell infiltration, and it provided an immunological perspective for developing personalized therapies.

## 1. Introduction

Colorectal cancer (CRC) is the third most common cancer and the second major cause of cancer death worldwide in 2020 [1]. The cornerstones of therapy for CRC include surgery for primary and metastatic disease, neoadjuvant radiotherapy for rectal cancer, and auxiliary chemotherapy for phase III/IV and high-risk phase II colon cancer [2, 3]. Nevertheless, these new treatment options have limited influence on cure rates and long-term survival. During the past two decades, the interconnections between patient prognosis and therapeutic response as well as the molecular mechanisms underlying the development of CRC have become increasingly apparent. In this regard, developing novel prognostic gene signatures for CRC is urgently required.

Programmed cell death includes apoptosis, necroptosis, ferroptosis, and pyroptosis [4]. Among the intrinsic forms of cell death, pyroptosis has recently received increasing attention. Pyroptosis is initiated by the gasdermin (GSDM) family and characterized by cell swelling and plasma membrane bubbling accompanied by inflammatory and immune responses [5]. Except for PJVK, the members of the GSDM superfamily (GSDMA, GSDMB, GSDMC, GSDMD, GSDME, and PJVK) harbour an N-terminal pore-generating domain and a C-terminal repressor domain as well as a linker connecting them [6]. The association between pyroptosis and cancer is complicated, and various tissues and genetic backgrounds exhibit varied effects of pyroptosis on cancer. On the one hand, the occurrence and growth of tumours can be inhibited by pyroptosis [7]; on the other hand, as a type of proinflammatory death, pyroptosis can generate a proper microenvironment for tumour cell development and thus drive tumour development [8]. Therefore, it is essential to identify the effect of pyroptosis on the prognosis of CRC.

Currently, some studies have reported prognostic gene signatures associated with pyroptosis in lung adenocarcinoma [9], ovarian cancer [10], gastric cancer [11], and CRC [12–16] based on data obtained from public databases. Our study provides a new method to identify a pyroptosis-associated prognostic gene signature for CRC. The above articles excluded genes with no significance based on the Cox regression model, which may ignore valuable information. In the current study, a prognostic model based on least absolute shrinkage and selection operator (LASSO) analysis was built using pyroptosis-associated genes without Cox regression model screening. We also determined the association of pyroptosis with the tumour immune microenvironment in CRC. Our findings may offer extra evidence on prognostic biomarkers and therapeutic targets for CRC.

We present the following article in accordance with the TRIPOD reporting checklist.

## 2. Methods

### 2.1. Patient Cohorts and Pyroptosis-Related Genes

UCSC Xena provided the Cancer Genome Atlas (TCGA) database (https://xena.ucsc.edu/) as a training cohort. The GSE39582 and GSE87211 databases were downloaded from the Gene Expression Omnibus (GEO) database (https://www.ncbi.nlm.nih.gov/gds/?term=) for validation. The extraction of pyroptosis-associated genes was performed from curated gene sets in Gene Set Enrichment Analysis (GSEA) (https://www.gsea-msigdb.org/gsea/msigdb). Protein-protein interaction (PPIs) network functional enrichment analysis was conducted in STRING (https://cn.string-db.org/cgi/input.pl).

### 2.2. Pyroptosis-Related Signature in CRC

All of the pyroptosis-related genes extracted from GSEA were included in the LASSO regression model in the TCGA database. LASSO was performed to identify the key signatures and the related parameters using the glmnet R package.

(1) $$\begin{array}{c} \text{Risk score}=\sum \left(\text{gene Expression}*\text{gene coefficient}\right). \end{array}$$

### 2.3. Genetic Mutations in Pyroptosis-Related Genes

Information on genetic mutations was obtained from cBioPortal (https://www.cbioportal.org/).

### 2.4. Kyoto Encyclopedia of Genes and Genomes (KEGG), Gene Ontology (GO) Analysis, and GSEA

Sangerbox tools, a free online platform for information analysis, were employed to perform KEGG, GO analysis, and GSEA. Briefly, samples were categorized into high- and low-risk groups, and GSEA was performed to identify the possible mechanisms associated with pyroptosis. Here, |NES| >1, NOM *p* value <0.05, and FDR *q*-value <0.25 were used as the thresholds of significance. The R software package clusterProfiler was applied for GO and KEGG functional enrichment analyses to identify pathway enrichment between the low-risk and high-risk groups.

### 2.5. Estimation of Tumour Microenvironment

Cell-Type Identification by Estimating Relative Subsets of RNA Transcripts (CIBERSORT) and MCPcounter was employed to forecast immune cell infiltration. The relative proportion of 22 immune cells was estimated with CIBERSORT, and MCPcounter scores evaluated the composition of 8 immune cells based on another algorithm. Then, correlation analysis was performed using Pearson's test.

### 2.6. Statistical Analysis

The survival time difference was measured by Kaplan–Meier analysis. The “time ROC” package in R was adopted to perform receiver operator characteristic (ROC) analyses. A *p* value <0.05 was considered statistically significant, and all *p* values were two-tailed.

## 3. Results

### 3.1. Identification of a Pyroptosis-Related Prognostic Signature

**Figure 1:**
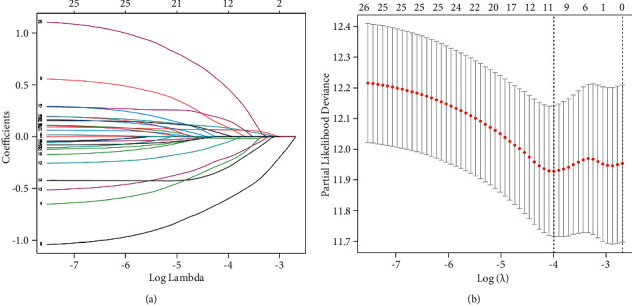
Screening of the pyroptosis-related prognostic signature by LASSO. (a) LASSO parameters of the pyroptosis-associated prognostic genes in TCGA. (b) The best fit profile was generated, and 12 pyroptosis-related genes were identified.

To date, 27 genes associated with pyroptosis have been identified from GSEA. To understand the function of the overall pyroptosis-related genes, univariate Cox analysis was not performed. None of the pyroptosis-related genes were excluded from the following analysis. LASSO regression analysis was performed using 27 pyroptosis-related genes, and 12 genes with the best capacity were identified using the LASSO regression model (Figures 1(a) and 1(b)). The description and parameters of the 12 pyroptosis-related genes are shown in Table 1.

**Figure 2:**
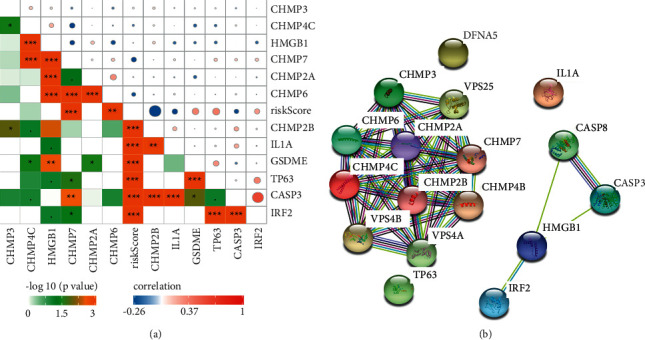
The correlation between the 12 pyroptosis-related genes. (a) Association between the risk score and the chosen signature genes in TCGA. Data were analysed using the Pearson coefficient. The colour represents the *p* value and coefficient. (b) The protein interaction network of pyroptosis-associated genes in TCGA.

The association network containing the 12 pyroptosis-associated genes and risk scores is displayed in Figure 2(a) (red: positive associations; blue: negative associations). For further exploration of the mutual effects of these pyroptosis-associated genes, PPIs between the 12 pyroptosis-related genes were analysed by using the STRING platform (Figure 2(b)).

Univariate and multivariate Cox analyses were performed using data from the TCGA cohort (Table 2). Univariate analysis indicated that risk score, age, and pathologic stage were related to the survival time of CRC patients. Multivariate analysis identified the risk score, age, and pathologic T stage as independent factors associated with CRC prognosis.

### 3.2. Landscape of Genetic Variation of Pyroptosis-Associated Genes in CRC

**Figure 3:**
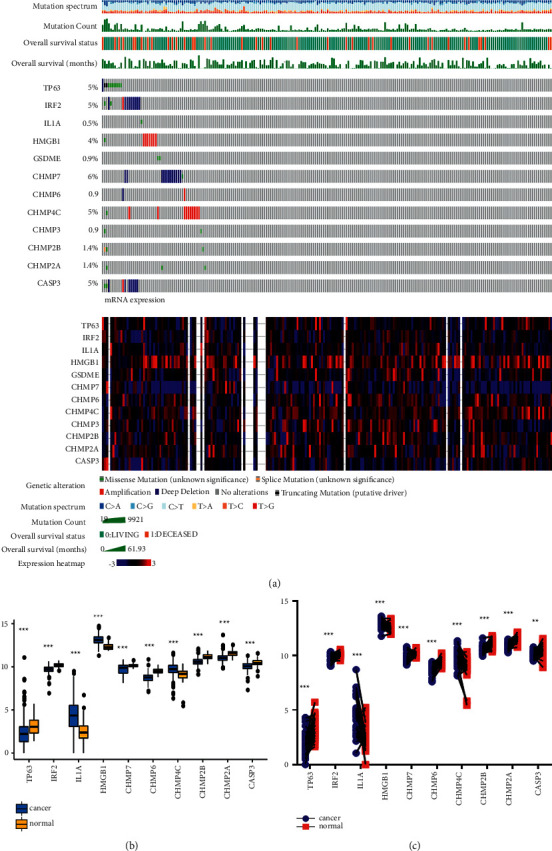
Genetic characteristics of pyroptosis-associated genes in CRC. (a) Genetic alteration, mutation spectrum, mutation count, overall survival time, and expression heatmap from the cBioPortal database. (b) Expression of the 12 pyroptosis-associated genes between CRC and normal tissues in TCGA. (c) Expression of the 12 pyroptosis-associated genes between CRC and paired normal tissues in TCGA.

At the genetic level, pyroptosis-related genes were altered in 51 (23%) of the 220 patients/samples (Figure 3(a)). Deep deletion was the most common variant classification, and C > T is ranked as the top mutation. Of these, TP63, IRF2, and CASP3 showed the highest frequency of mutations, and only 1 sample (approximately 0.5%) harboured IL1A missense mutations. Figure 3(a) also presents the mutation count, survival status, survival time, and expression heatmaps of these genes.

In the TCGA cohort, IL1A, HMGB1, and CHMP4C were enriched in CRC samples, whereas the remaining pyroptosis-related genes all showed decreased expression in CRC samples (Figure 3(b)). For more effective verification of our findings, the mRNA levels of pyroptosis-associated genes among 32 pairs of tumour tissues and normal nearby tissue samples were tested. Similarly, these pyroptosis-related genes were downregulated in tumours (Figure 3(c)).

### 3.3. The Prognostic Capacity of the Pyroptosis-Associated Gene Signature

**Figure 4:**
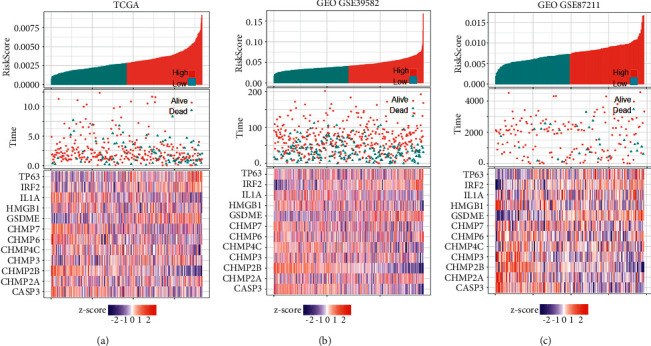
Overview of the pyroptosis-related gene signature. Allocation of risk markers, survival time, and expression heatmap of signature genes in the TCGA (a), GEO GSE39582 (b), and GEO GSE87211 (c) cohorts.

CRC patients were separated into high- and low-risk groups based on their median risk score. Figures 4(a)–4(c) show the risk score allocation, survival status, and gene profiles of the 12 genes in the training (TCGA) and verification (GSE39582 and GSE87211) cohorts. As the risk score increased, the patients' risk of death increased, and the survival time declined. The heatmaps showed that CHMP2A, CHMP3, GSDME, HMGB1, IRF2, and TP63 were overexpressed, whereas downregulation of CHMP2B, CASP3, CHMP4C, CHMP6, CHMP7, and IL1A was observed in high-risk cases.

**Figure 5:**
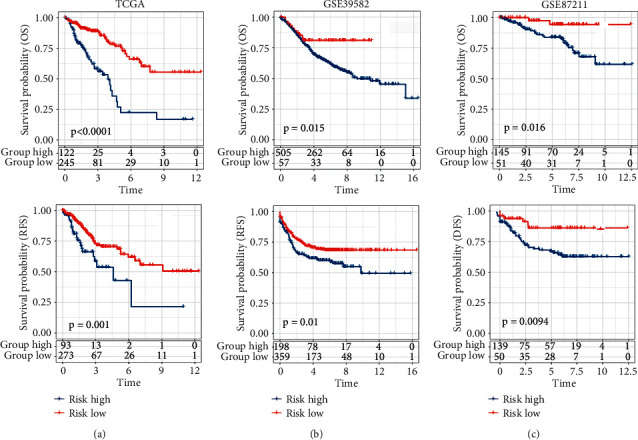
Kaplan–Meier curve of the pyroptosis-related gene signature. Overall, progression-free or relapse-free survival for CRC patients with a high or low pyroptosis-related risk score in the TCGA (a), GEO GSE39582 (b), and GEO GSE87211 (c) cohorts.

Next, the value of the risk score was further determined by forecasting the prognosis of patients. Patients were classified into high- and low-risk score groups based on the best cut-off value. According to the Kaplan–Meier curve, CRC patients with a high-risk score showed a worse total survival, progression-free survival, or relapse-free survival compared with those with a low-risk score (Figures 5(a)–5(c)). The number of patients at risk is displayed in the lower portion of each graph.

**Figure 6:**
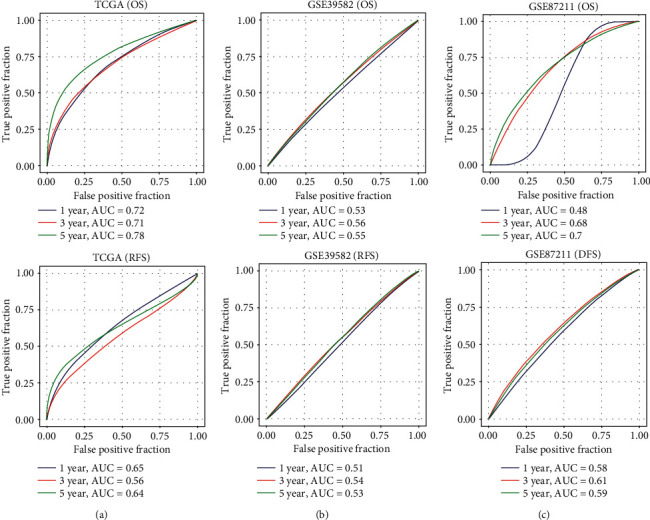
ROC curves on the basis of overall survival and progression-free or relapse-free survival of the risk score in the TCGA (a), GEO GSE39582 (b), and GEO GSE87211 (c) cohorts to evaluate the predictive capacity of the signature.

The sensitivity and specificity of the prognostic model were assessed with time-dependent ROC analysis, and area under curve (AUC) in the TCGA, GSE39582, and GSE87211 cohorts predicting overall survival reached 0.78, 0.55, and 0.7 for 5-year survival (Figures 6(a)–6(c)). AUC values predicting relapse-free survival or progression-free survival were also significant at >0.5 (Figures 6(a)–6(c)).

### 3.4. Functional Enrichment Analysis of Pyroptosis-Associated Genes

To clarify the function of the pyroptosis-related genes between the subgroups classified by the risk model, we identified 512 differentially expressed genes (DEGs) with an absolute value of log10 FDR <1 and |log2-fold change| <1 between the low- and high-risk groups in the TCGA cohort. The KEGG pathway analysis and GO enrichment analysis were then conducted based on these 512 DEGs.

**Figure 7:**
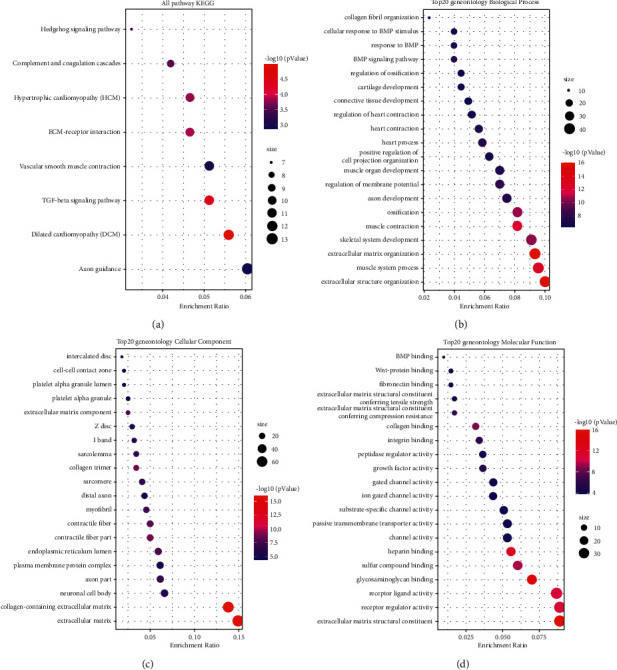
Functional enrichment analysis of pyroptosis-associated genes. KEGG (a) and GO (b–d) enrichment analyses of pyroptosis-related genes in patients with high or low-risk scores in TCGA.

We found that these DEGs were mostly involved in the ECM-receptor interaction, vascular smooth muscle contraction, TGF-beta signalling pathway, dilated cardiomyopathy (DCM), and axon guidance based on KEGG pathway analysis (Figure 7(a)). Moreover, GO analysis suggested that these DEGs were mainly correlated with extracellular matrix, extracellular structure organization, and extracellular matrix structural constituent (Figures 7(b)–7(d)).

**Figure 8:**
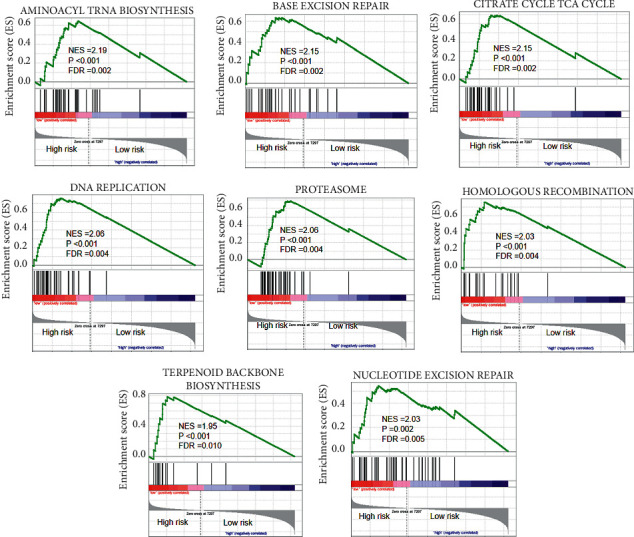
GSEA analyses of pyroptosis-related genes between high- and low-risk groups in TCGA.

Subsequently, GSEA was performed between the high- and low-risk groups. On the basis of the outcomes, the high-risk group expressed all enriched gene sets. These gene sets were involved in mechanisms associated with aminoacyl tRNA biosynthesis, base excision repair, citrate cycle TCA cycle, DNA replication, proteasome, homologous recombination, terpenoid backbone biosynthesis, and nucleotide excision repair (Figure 8).

### 3.5. Identification of the Association between Pyroptosis-Related Genes and the Tumour Immune Microenvironment

**Figure 9:**
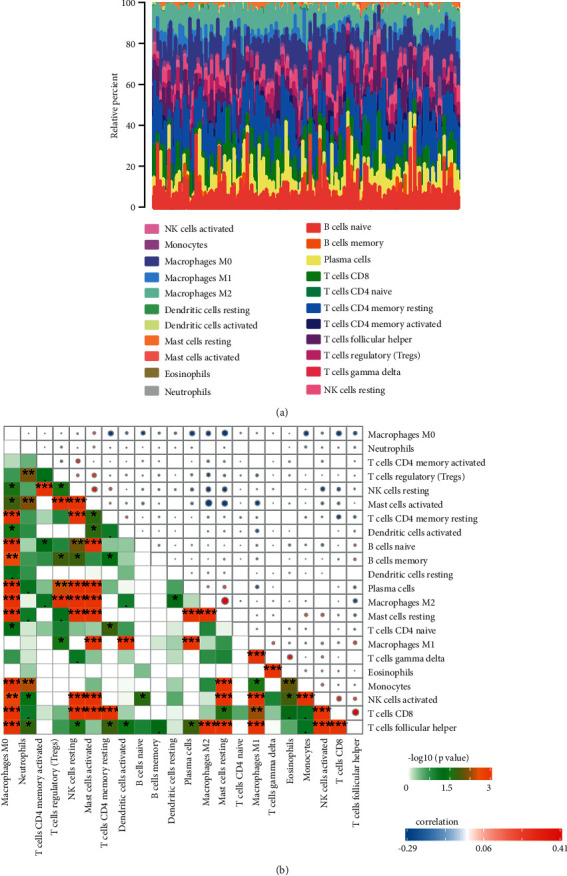
Overview of immune cells infiltrated by the CIBERSORT algorithm. (a) The relative percentage of tumour-infiltrating immune cells in CRC. (b) The association between tumour-infiltrating immune cells in CRC.

To better assess how pyroptosis-related genes interact with the immune microenvironment, the CIBERSORT algorithm was adopted, and integrated comparisons with the risk scores were conducted. Figures 9(a) and 9(b) show the relative content allocation of tumour-infiltrating immune cells in the TCGA cohort and the association between tumour-infiltrating immune cells.

**Figure 10:**
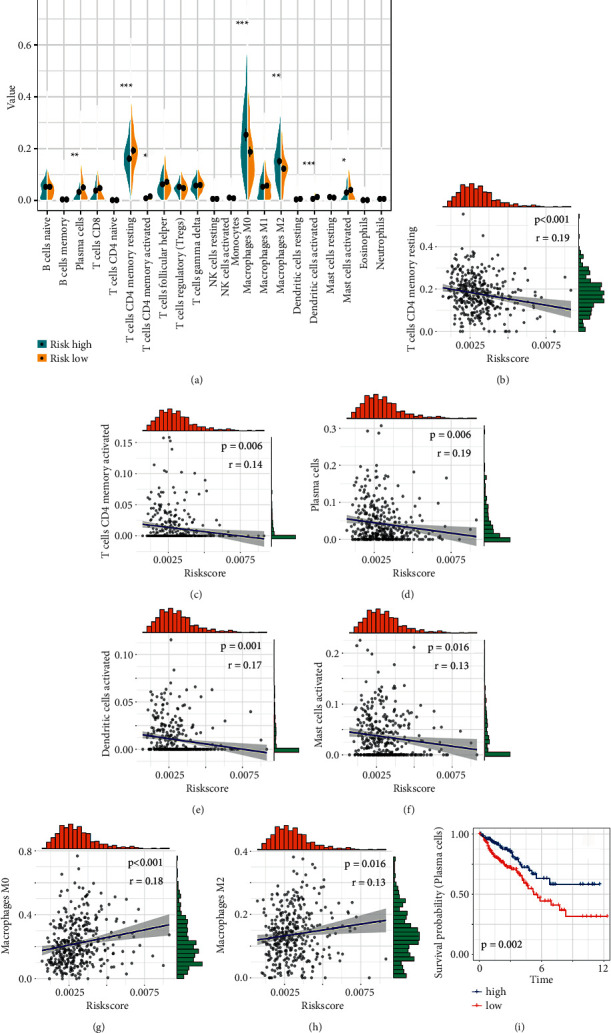
Association between immune cells and pyroptosis-related risk score. (a) The variation in infiltrating immune cells between the high- and low-risk groups. (b–h) The correlation between the risk score and M2 macrophages, M0 macrophages, plasma cells, resting memory CD4 T cells, activated memory CD4 T cells, activated dendritic cells, and activated mast cells. (i) Kaplan–Meier curve of plasma cells in TCGA.

Next, whether the risk score would have guiding value for clinical treatment, especially immunotherapy, was investigated. The infiltration of tumor microenvironment (TME) cells was analysed in patients with various risk scores (Figure 10(a)). Patients in the low-risk group showed a higher percentage of antitumoural immune cells, such as plasma cells, CD4 T cells, activated dendritic cells (DC), and activated mast cells. Higher scores were closely related to M2 macrophages and M0 macrophages. Similarly, further analysis showed that the risk score was positively related to M2 macrophages and M0 macrophages but negatively related to plasma cells, CD4 T cells, activated dendritic cells, and activated mast cells (Figures 10(b)–10(h)). Furthermore, Kaplan–Meier analysis indicated that plasma cells conferred a significant survival advantage (Figure 10(i)).

**Figure 11:**
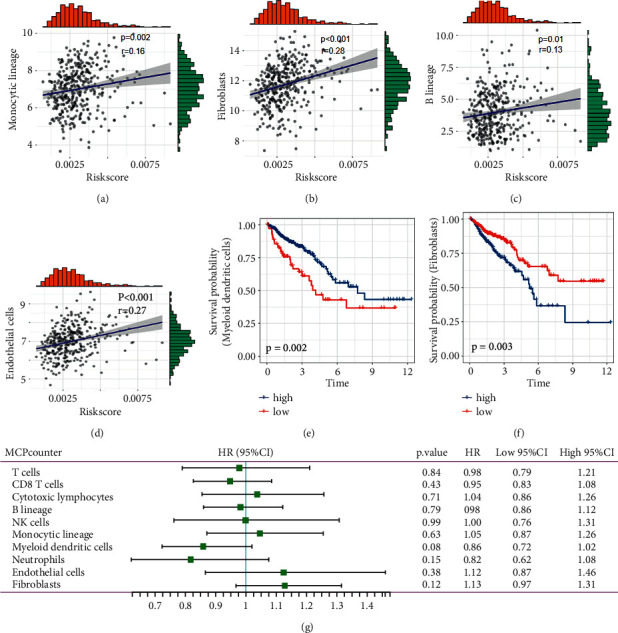
Association between immune cells and pyroptosis-related risk score using MCPcounter. (a–d) The association between risk score and monocyte lineage, fibroblasts, B lineage, and endothelial cells. (e, f) Kaplan–Meier curve of myeloid dendritic cells and fibroblasts in TCGA.

The MCP-counter score showed the association between the risk score and the monocytic lineage, fibroblasts, B lineage, and endothelial cells in the TCGA cohort (Figures 11(a)–11(d)). Conversely, Kaplan–Meier analysis (Figures 11(e) and 11(f)) and univariate Cox regression (Figure 11(g)) highlighted that CRC patients benefited from myeloid dendritic cells, whereas fibroblasts impaired CRC patient prognosis.

## 4. Discussion

This research established a pyroptosis-related prognostic signature for CRC patients. TCGA cohort data were assessed using the LASSO regression model, and a 12-gene signature associated with the outcome of CRC was produced. ROC curve and Kaplan–Meier analyses among patients with regard to high- and low-risk scores were validated in the GEO cohort. Genetic variation analysis showed that TP63, IRF2, and CASP3 presented the highest frequency of mutations. In the following functional analysis, KEGG, GO, and GSEA results indicated that the high-risk groups were related to extracellular matrix, TGF-beta signalling pathway, and DNA replication. Immune cell infiltration data demonstrated that the low-risk group exhibited more activated immune cell infiltration, which may result in a better response to immunotherapy. This work will promote future pyroptosis studies in CRC.

Consistent with our observations, other researchers also identified pyroptosis-associated models and characterized tumour microenvironment infiltration in CRC. Generally, genes with no significance were excluded using the Cox regression model before lasso analysis. However, the Cox regression model failed to take advantage of genes that had important biological functions without statistical significance. Many valuable pieces of information, such as biological characteristics, have been ignored. In our study, the threshold of Cox analysis was abolished. Based on the overall trend of the dataset, our study provided a more reasonable pyroptosis-related prognosis signature.

We identified a 12 pyroptosis-associated gene signature for the prognosis of colorectal cancer, including CAS P3, CHMP2A, CHMP2B, CHMP3, CHMP4C, CHMP6, CHMP7, GSDME, HMGB1, IL1A, IRF2, and TP63. Activated caspase 3 could specifically cleave GSDME [17], which generated pores in the plasma membrane and induced pyroptosis. In certain cells, the switch from apoptosis to pyroptosis could be induced by knocking out GSDME [17]. GSDME expression was silenced in colorectal, gastric, and breast cancer [18], indicating that GSDME may be a tumour suppressor gene. Furthermore, GSDME-induced pyroptosis was an underlying mechanism involved in the resistance to the toxicity of chemotherapy drugs [17]. CHMP2A, CHMP2B, CHMP3, CHMP4C, CHMP6, and CHMP7 are endosomal sorting complexes necessary for transport (ESCRT)-III subunit [19]. ESCRT-III plays a key role in the degradation of transmembrane proteins in lysosomes, midbody abscission during cytokinesis, and retroviral budding. In the pyroptosis process, ESCRT-III is required for damaged plasma membrane repair [20]. Given that EGFR is a cell membrane protein, inactivation of the ESCRT-III machinery impairs EGFR degradation [21], which may induce tumorigenesis. Furthermore, CHMP4C has been frequently overexpressed in human cancer tissue [22]. As a response to damage-associated molecular patterns (DAMPs), the nuclear-associated protein HMGB1 and cytokine IL1A are released following exposure to inflammatory stimuli [23]. HMGB1 overexpression was confirmed in melanoma, colon cancer, prostate cancer, pancreatic cancer, and breast cancer [24]. During pyroptosis, caspase-1, which is activated by inflammasomes, promotes the secretion of HMGB1 and IL1A [25, 26]. Subsequently, HMGB1 and IL1A promoted the expression of vascular endothelial growth and tumorigenesis. IRF-2 is a member of the interferon regulatory factor (IRF) family, which was originally identified based on its effects on innate and adaptive immunity. IRF-2 targeted the interferon-stimulated response element and was involved in the regulation of the interferon system [27]. IRF2, which is overexpressed in pancreatic cancer [28] and oesophageal squamous cell carcinoma [29], acts as an oncogene. IRF2 sequentially activates caspase-4 and GSDMD to induce pyroptosis [30]. TP63 is a member of the p53 family and exhibits diverse biological functions, such as cellular proliferation, differentiation, stem cell maintenance, cell death and survival, DNA damage response, and metabolism. Furthermore, multiple isoforms of TP63 played opposite roles in different human tumours [31, 32]. Numerous studies have confirmed that TP63 can induce apoptosis and DNA damage [33]. In CRC, knockdown of TP63 could cleave GSDME to induce pyroptosis [34].

More recently, immunotherapy and the tumour microenvironment have attracted increasing attention. Immune cells, such as T lymphocytes and B lymphocytes, infiltrated the tumour microenvironment and influenced tumour progression. In our study, pyroptosis risk scores were negatively related to plasma cells, CD4 T cells, activated dendritic cells, and activated mast cells and positively related to M2 macrophages and M0 macrophages. Generally, M2 macrophages secrete cytokines, such as IL-10 and TGF-*β*, which suppress the immune response and promote tumorigenesis [35]. Conversely, high proportions of infiltrating CD4 T cells and dendritic cells, which are optimally equipped antigen-presenting cells (APCs), were associated with a favourable prognosis. Upon activation by tumour antigens, DCs can internalize, process, and later show processed epitopes to T cells and cause cytotoxic T lymphocyte (CTL) immune responses [36]. CD4 T cells were mainly helper T cells (Th cells) in our study. Th cells play a role in antitumour immunity by assisting CD8 effector T cells and functioning cytotoxic T cells [37, 38].

## 5. Conclusion

In our study, a new, reasonable pyroptosis-associated prognostic signature for CRC was observed and an extensive regulatory mechanism by which they affect the prognosis was revealed. Genetic variation analysis presented the high mutation frequency of pyroptosis-related genes. Moreover, we identified that immune cell infiltration in the low-risk group contributes to CRC patient prognosis. Our findings highlight the crucial implications of pyroptosis-related genes, which may be developed as a precise indicator for individualized clinical prognostication.

## Figures and Tables

**Table 1:** The list of of the 12 pyroptosis-related genes.

Gene symbol	Description	Coefficient
CASP3	Caspase 3	−0.2358855894
CHMP2A	Charged multivesicular body protein 2A	0.1535683588
CHMP2B	Charged multivesicular body protein 2B	−0.5961559646
CHMP3	Charged multivesicular body protein 3	0.0867141558
CHMP4C	Charged multivesicular body protein 4C	−0.0477902078
CHMP6	Charged multivesicular body protein 6	−0.1967124331
CHMP7	Charged multivesicular body protein 7	−0.2900477106
GSDME	Gasdermin E	0.0820987643
HMGB1	High mobility group box 1	0.0099448303
IL1A	Interleukin 1 alpha	−0.0173466610
IRF2	Interferon regulatory factor 2	0.4575734946
TP63	Tumor protein p63	0.0662656359

Risk score = (−0.2359 ^*∗*^CASP3) + (0.1536 ^*∗*^ CHMP2A) + (−0.5962 ^*∗*^ CHMP2B) + (0.0867 ^*∗*^ CHMP3) + (−0.0478 ^*∗*^ CHMP4C) + (−0.1967 ^*∗*^ CHMP6) + (−0.29 ^*∗*^ CHMP7) + (0.0821 ^*∗*^ GSDME) + (0.0099 ^*∗*^ HMGB1) + (−0.0173 ^*∗*^ IL1A) + (0.4576 ^*∗*^ IRF2) + (0.0663 ^*∗*^ TP63).

**Table 2:** The univariate and multivariate cox regression of between high- and low-risk group in TCGA.

Variable	Univariate cox analysis		Multivariate cox analysis	
	HR (95% CI)	*p* value	HR (95% CI)	*p* value
Riskscore	3.278 (2.119–5.071)	<0.001	2.621 (1.679–4.093)	<0.001
Age	1.036 (1.015–1.059)	<0.001	1.046 (1.023–1.07)	<0.001
Gender	1.068 (0.657–1.738)	0.79	0.924 (0.561–1.522)	0.756
Pathologic stage	2.155 (1.624–2.861)	<0.001	1.896 (0.783–4.589)	0.156
Pathologic T	3.292 (1.93–5.615)	<0.001	2.013 (1.034–3.918)	0.04
Pathologic N	1.907 (1.428–2.548)	<0.001	1.021 (0.598–1.742)	0.941
Pathologic M	3.685 (2.169–6.26)	<0.001	1.361 (0.404–4.587)	0.619
MSI	0.959 (0.697–1.318)	0.795	0.773 (0.548–1.092)	0.144

## Data Availability

The data used to support the findings of this study are available from the corresponding author upon request.
